# Integration of BOLD-fMRI and DTI into radiation treatment planning for high-grade gliomas located near the primary motor cortexes and corticospinal tracts

**DOI:** 10.1186/s13014-015-0364-1

**Published:** 2015-03-08

**Authors:** Minglei Wang, Hui Ma, Xiaodong Wang, Yanhong Guo, Xinshe Xia, Hechun Xia, Yulin Guo, Xueying Huang, Hong He, Xiaoxiong Jia, Yan Xie

**Affiliations:** Department of Radiology, General Hospital of Ningxia Medical University, Yinchuan, China; Ningxia Key Laboratory for Cerebrocranial Diseases, Yinchuan, China; Department of Neurosurgery, General Hospital of Ningxia Medical University, Yinchuan, China; Department of Radiation Oncology, General Hospital of Ningxia Medical University, Yinchuan, China; Department of Radiology, Xi’an NO.1 Hospital, Xi’an, China; Tissue Organ Bank & Tissue Engineering Centre, General Hospital of Ningxia Medical University, Yinchuan, Ningxia China; Tissue Repair and Regeneration Program, Institute of Health and Biomedical Innovation, Queensland University of Technology, Kelvin Grove, QLD Australia

**Keywords:** High-grade gliomas, Blood oxygen level dependent functional magnetic resonance imaging (BOLD-fMRI), Diffusion tensor imaging (DTI), Three-dimensional conformal radiation treatment (3DCRT), Intensity-modulated radiation therapy (IMRT), Radiation treatment planning

## Abstract

**Background:**

The main objective of this study was to evaluate the efficacy of integrating the blood oxygen level dependent functional magnetic resonance imaging (BOLD-fMRI) and diffusion tensor imaging (DTI) data into radiation treatment planning for high-grade gliomas located near the primary motor cortexes (PMCs) and corticospinal tracts (CSTs).

**Methods:**

A total of 20 patients with high-grade gliomas adjacent to PMCs and CSTs between 2012 and 2014 were recruited. The bilateral PMCs and CSTs were located in the normal regions without any overlapping with target volume of the lesions. BOLD-fMRI, DTI and conventional MRI were performed on patients (Karnofsky performance score ≥ 70) before radical radiotherapy treatment. Four different imaging studies were conducted in each patient: a planning computed tomography (CT), an anatomical MRI, a DTI and a BOLD-fMRI. For each case, three treatment plans (3DCRT, IMRT and IMRT_PMC&CST) were developed by 3 different physicists using the Pinnacle planning system.

**Results:**

Our study has shown that there was no significant difference between the 3DCRT and IMRT plans in terms of dose homogeneity, but IMRT displayed better planning target volume (PTV) dose conformity. In addition, we have found that the Dmax and Dmean to the ipsilateral and contralateral PMC and CST regions were considerably decreased in IMRT_PMC&CST group (*p* < 0.001).

**Conclusions:**

In conclusion, integration of BOLD-fMRI and DTI into radiation treatment planning is feasible and beneficial. With the assistance of the above-described techniques, the bilateral PMCs and CSTs adjacent to the target volume could be clearly marked as OARs and spared during treatment.

## Background

Gliomas, which contain oligodendroglia, astrocytic and ependymal lesions are the most common primary intracranial tumors. High-grade gliomas, which make up 35 to 45% of all newly diagnosed primary brain tumors worldwide, have a very poor prognosis [1]. The three-dimensional conformal radiotherapy (3DCRT) has been considered as the standard therapy for patients with high-grade gliomas and intensity-modulated radiotherapy (IMRT) is becoming increasingly used to improve dose conformity and spare critical normal tissues. However, the risk of radiation-induced brain injury increases with the increase of radiation dose [2-4]. The strenuous endeavor has been made to diminish radiation complications.

With the assistance of conventional magnetic resonance imaging (MRI) and planning computed tomography (CT) data, many critical intracranial structures, such as lens, optic nerves and optic chiasm are well demarcated. However, it is difficult to accurately locate eloquent cortices and fiber connections in the white matter of the brain by routine neuroimaging. Excessive irradiation of eloquent cortices and white matter fiber tracts is unavoidable. Blood oxygen level dependent functional magnetic resonance imaging (BOLD-fMRI) and diffusion tensor imaging (DTI) have recently been used to identify the primary motor cortexes (PMCs) and corticospinal tracts (CSTs). These imaging techniques have been implemented in modern neuronavigation systems and used to guide the surgical removal of critically located intracranial lesions [5,6]. The purpose of our study was to evaluate whether the incorporation of BOLD-fMRI and DTI data into the 3D treatment planning process could spare the healthy brain and sensitive parts of the brain from high doses of radiation.

## Methods

Ethical Approval was obtained from the General Hospital of Ningxia Medical University Review Board and written informed consent was obtained from patients. The study was conducted with strict adherence to the Declaration of Helsinki Principles.

### Subjects

A total of 20 patients with high-grade gliomas adjacent to PMCs and CSTs between May 2012 and February 2014 were recruited from the General Hospital of Ningxia Medical University, China. Eleven male patients and 9 female patients aged from 24 to 66 year-old were enrolled in this study.

The glioma tissues in our study included 14 astrocytomas (WHO Grade III) and 6 glioblastomas (WHO Grade IV). The bilateral PMCs and CSTs were located in the normal regions without any overlapping with target volume of the lesions. BOLD-fMRI, DTI and conventional MRI were performed on patients (Karnofsky performance score ≥ 70) before radical radiotherapy treatment.

### Data acquisition and analysis

Four different imaging studies were conducted in each patient: a planning CT for radiosurgery treatment and target tracking during radiation therapy treatment delivering; an anatomical MRI to deliver a complete set of morphological MR data; a DTI to provide white matter tractography and a BOLD- fMRI to provide brain activation maps. Axial CT images (3-mm slice thickness) were taken by a wide-bore Siemens Somatom Sensation Open CT scanner (Siemens, Germany). MRI volumes were acquired using a Signal HDx 3.0 T MRI scanner (General Electric Company, USA).

BOLD fMRI data were obtained using fat-saturated single-shot gradient echo planar imaging (EPI) (TE = 35 ms, TR = 3,000 ms, acquisition matrix = 64 × 64 pixels, FOV = 240 mm × 240 mm^2^, flip angle = 90°, NEX = 1, 3 mm thickness). A block design paradigm (5 cycles, 30 sec on and 30 sec off) was utilized. Functional areas relevant to each treatment region were probed by Somatosensory tasks (finger tapping with audio cue). Following the acquisition of the functional data, gadolinium-enhanced high-resolution images were acquired (TR/TE = 450/14, flip angle = 90°, matrix = 256 × 256, FOV = 240 mm × 240 mm^2^, and slice thickness 3 mm skip 0 mm). After the images were taken, data were transferred to the Matlab workstation for analysis. The DTI data acquisition sequence was a spin echo-echo planar imaging (SE-EPI) sequence with TR = 10,000 ms, TE = 98.8 ms, acquisition matrix = 128 × 128 pixels; FOV = 240 mm × 240 mm^2^; slice thickness = 3.0 mm. Diffusion-weighted imaging with b factor of 1,000 mm^2^/s was taken along 25 noncollinear directions. The acquisition time of DTI sequence was 280 seconds. DTI data was analyzed on-line by the advantage workstation of the MR scanner (AW 4.4). For CST analysis, a seed region of interest (ROI) and a target ROI were placed on the posterior limb of the internal capsule and pons (anterior blue portion on the color map). Fiber tracking employed fractional anisotropy (FA) threshold of 0.2 and a tract angular change of 30°. The color-coded FA maps were merged with the anatomical MRI images. Regions of interest were drawn on the fused FA maps.

**Figure 1:**
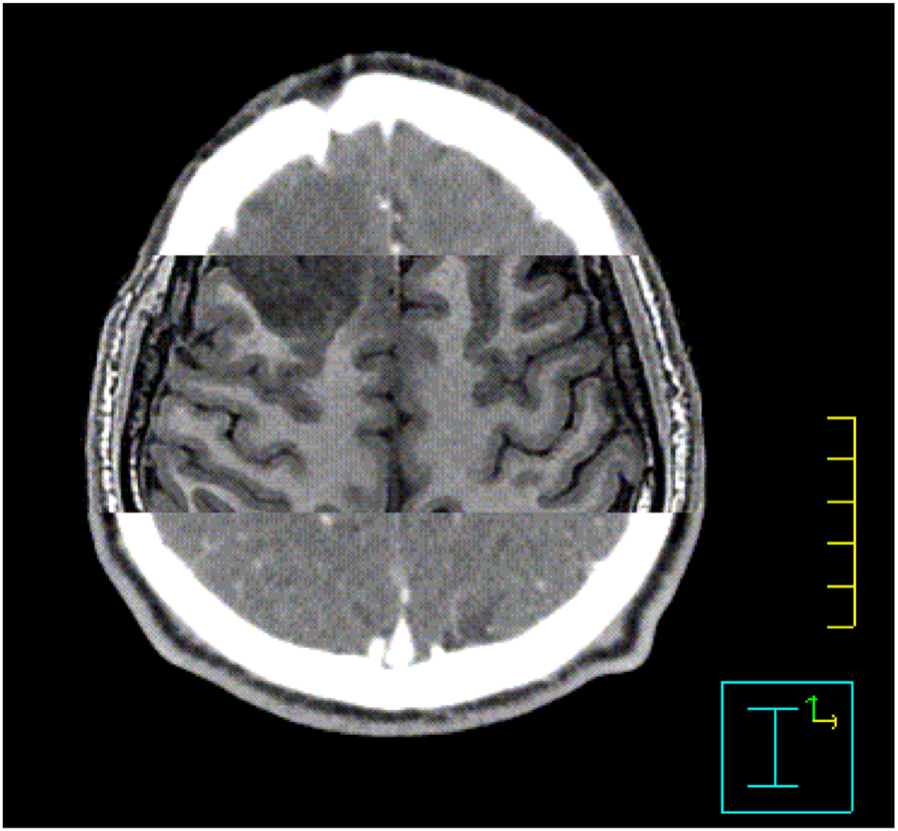
**T1-weighted MR imaging and the corresponding axial CT after registration.**

The fused fMRI activation maps and the white matter tracts overlaid on the anatomical MRI volume were exported as separate grayscale dicom images and loaded onto Pinnacle planning system software version 9.2 (Philips Medical Systems, Netherlands). The anatomical MRI images were registered with the CT volume for each patient. Figure 1 shows the anatomical MRI images were registered with the corresponding axial CT planes for a glioma case.

### Treatment planning

The target and organs at risk (OARs), i.e., optic nerves, optic chiasm and brain stem were precisely described using CT/anatomical MRI images. Both eyes were protected to avoid beam damage during treatment planning. The PMCs and the CSTs situated near the target were defined by a radiologist and a neurosurgeon, using the tractography images and the fused activation maps. Gross tumor volume (GTV) was described as the operative cavity with any remaining contrast-enhancing tissue on T1-weighted magnetic resonance imaging or as unresected enhancing tumor. The initial clinical target volume (CTV1) was defined as the T2 hyper intensity area (edema) with a 20 mm expansion. An initial planning target volume (PTV1) was created by adding a 30 mm expansion to the CTV1 to account for setup uncertainties. A second clinical target volume (CTV2) was defined as the contrast enhancement region in T1 with an additional 25 mm margin. A PTV2 was generated by adding a 30 mm expansion to the CTV2.

**Figure 2:**
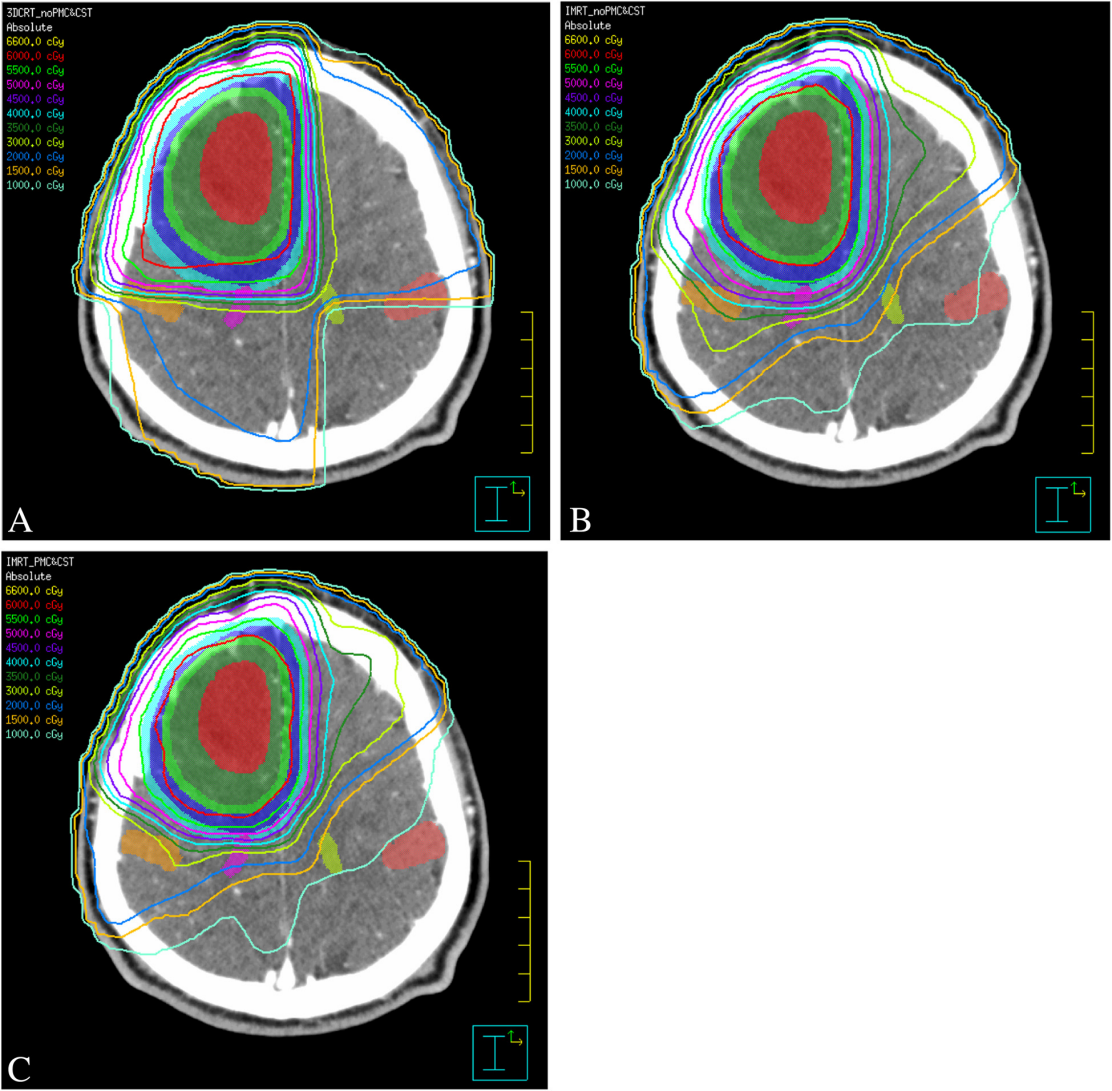
**Axial isodose distribution in a patient with high-grade glioma.** The **A**, **B** and **C** show the dose distributions for 3DCRT, IMRT and IMRT_ PMC&CST, respectively. (orange) ipsilateral PMC (red square symbol) contralateral PMC (pink square symbol) ipsilateral CST (green square symbol) contralateral CST.

For each case, three treatment plans were developed by 3 different physicists using the Pinnacle planning system. The first physicist created conventional 3DCRT plans (3DCRT) and the PMCs and the CSTs situated near the target were not taken in account by the physicist. The target and standard morphological OARs were considered in this plan. The second physicist developed IMRT plans (IMRT) and the PMCs and the CSTs situated near the target were not considered by the physicist. The third physicist developed IMRT plans (IMRT_PMC&CST), and the PMCs and the CSTs situated near the target were considered. Figure 2 shows the axial isodose distribution of a patient with high-grade glioma.

The treatment plans met the requirement that at least 95% of the PTV receives the prescribed dose. Cumulative doses to the lenses, optic nerves, optic chiasm, and brainstem were limited to a maximum dose of 54 Gy for the last three structures and as low as practically achievable for the former. For conventional 3DCRT treatment, the prescribed dose was 50 Gy to the PTV1, immediately followed by 10 Gy to the PTV2, with a total cumulative dose of 50 Gy to the PTV1 and 60 Gy to the PTV 2 both at 2 Gy per fraction. For IMRT plans, the prescribed dose was 50 Gy to the PTV1 and 60 Gy to the PTV2, which were delivered concurrently over 30 daily fractions, with a fractional dose of 2 Gy to the PTV2.

### Comparison criteria for the radiation treatment plans

The dose volume histograms (DVH) data were obtained from each patient. The dose coverage was analyzed according to the mean dose (Dmean), maximum dose (Dmax), conformity index (CI) and homogeneity index (HI). The CI was defined as follows [7]: CI = VRI/PTV, where VRI represents the volume covered by the prescription dose. A CI value of 1.0 indicates that the volume of the prescription isodose surface is equal to that of the PTV. The HI was defined as follows [8]: HI = (D2 − D98)/D50, where Dx% represents the dose delivered to x% of the PTV. Lower HI values indicate a more homogeneous target dose. OARs (e.g., brainstem, optic chiasm, optic nerves, and lenses) and PMCs and CSTs were compared based on the values of Dmax and Dmean.

### Statistical analyses

The comparison of parameters between different plans was analyzed by the paired two-tailed Student *t* test. Differences were considered statistically significant at *p* < 0.05.

## Results

### Comparison of target volume coverage and OAR sparing between 3DCRT and IMRT

Parameters related to dose coverage planning for 3DCRT and IMRT are presented in Tables 1 and 2. The results indicated that there was no significant difference between the 3DCRT and IMRT plans in terms of dose homogeneity, but IMRT displayed better PTV dose conformity. Regarding the comparison of PTV1 Dmax, PTV1 Dmean, PTV2 Dmax and PTV2 Dmean, there was no significant difference between the 3DCRT and IMRT plans. The dosimetric details of brainstem, optic chiasm, optic nerves, and lenses revealed no significant differences between the two plans and all of these organs were strictly maintained within the dose limitations. The Dmax and Dmean of PMCs and CSTs were observed in both 3DCRT and IMRT plans (Table 3); however no significant difference was found between the two plans.

**Table 1:** **Comparison of target volume coverage between 3DCRT and IMRT**

	**3DCRT**	**IMRT**	***t***	***p***
PTV1 (50 Gy)				
D_max_ (Gy)	65.06 ± 0.46	64.88 ± 0.66	1.461	0.160
D_mean_ (Gy)	59.78 ± 0.77	59.91 ± 0.85	−0.551	0.588
CI	1.219 ± 0.054	1.071 ± 0.025	10.492	<0.001*
HI	0.210 ± 0.008	0.213 ± 0.012	−1.177	0.254
PTV2 (60 Gy)				
D_max_ (Gy)	65.06 ± 0.46	64.88 ± 0.66	1.461	0.160
D_mean_ (Gy)	62.46 ± 0.39	62.36 ± 0.53	1.301	0.209
CI	1.178 ± 0.082	1.055 ± 0.049	5.552	<0.001*
HI	0.086 ± 0.022	0.082 ± 0.016	0.809	0.429

*Significant difference.

**Table 2:** **Comparison of OAR sparing between 3DCRT and IMRT**

	**3DCRT (Gy)**	**IMRT (Gy)**	***t***	***p***
Ipsilateral lens D_max_	1.99 ± 1.00	2.67 ± 1.67	−1.938	0.068
Ipsilateral lens D_mean_	1.52 ± 0.75	1.98 ± 1.45	−1.454	0.162
Contralateral lens D_max_	1.71 ± 0.74	2.38 ± 1.97	−1.885	0.075
Contralateral lens D_mean_	1.34 ± 0.55	1.84 ± 1.69	−1.585	0.129
Ipsilateral optic nerve D_max_	12.54 ± 17.85	12.32 ± 13.38	0.156	0.877
Ipsilateral optic nerve D_mean_	8.55 ± 11.99	8.57 ± 9.40	−0.014	0.989
Contralateral optic nerve D_max_	7.55 ± 8.94	7.02 ± 7.68	1.025	0.318
Contralateral optic nerve D_mean_	5.14 ± 5.92	4.59 ± 4.76	1.364	0.188
Optic chiasm D_max_	12.53 ± 15.50	12.76 ± 13.53	−0.241	0.812
Optic chiasm D_mean_	9.22 ± 11.64	8.28 ± 8.70	1.060	0.302
Brainstem D_max_	14.78 ± 14.77	14.04 ± 11.62	0.523	0.607
Brainstem D_mean_	7.09 ± 8.31	6.52 ± 6.99	0.711	0.486

**Table 3:** **Comparion of radiation dose between 3DCRT and IMRT**

	**3DCRT (Gy)**	**IMRT (Gy)**	***t***	***p***
Ipsilateral PMC D_max_	46.50 ± 8.65	46.54 ± 7.77	−0.050	0.960
Ipsilateral PMC D_mean_	28.45 ± 7.78	27.67 ± 8.06	1.429	0.169
Contralateral PMC D_max_	24.86 ± 9.89	21.40 ± 10.94	1.542	0.140
Contralateral PMC D_mean_	14.73 ± 6.02	14.11 ± 7.57	0.390	0.701
Ipsilateral CST D_max_	51.26 ± 4.24	50.61 ± 4.72	1.801	0.088
Ipsilateral CST D_mean_	36.51 ± 6.63	36.06 ± 7.57	0.893	0.383
Contralateral CST D_max_	35.64 ± 10.15	36.11 ± 10.05	−0.859	0.401
Contralateral CST D_mean_	20.97 ± 7.43	18.90 ± 7.45	1.454	0.162

### Comparison of target volume coverage and OAR sparing between IMRT and IMRT_PMC&CST

Treatment plan parameters are shown in Tables 4 and 5. According to the data presented, both PTV1 and PTV2 (Dmax, Dmean, CI and HI) were analyzed and showed no significant differences between two groups. The Dmax and Dmean to these conventional OARs (e.g., brainstem, optic chiasm, optic nerves, and lenses) showed no significant differences between the two plans. The Dmax to the ipsilateral and contralateral PMC and CST regions was considerably decreased by 28.7%, 24.5%, 20.2% and 37.6%, respectively. The Dmean to the ipsilateral and contralateral PMC and CST regions was considerably decreased by 27.8%, 30.4%, 23.1% and 33.4%, respectively (Table 6).

**Table 4:** **Comparison of target coverage between IMRT and IMRT_PMC&CST**

	**IMRT**	**IMRT_PMC&CST**	***t***	***p***
PTV1 (50 Gy)				
D_max_ (Gy)	64.88 ± 0.66	65.04 ± 0.70	−0.806	0.430
D_mean_(Gy)	59.91 ± 0.85	59.85 ± 0.68	0.633	0.534
CI	1.071 ± 0.025	1.073 ± 0.024	−1.077	0.295
HI	0.213 ± 0.012	0.209 ± 0.016	0.911	0.374
PTV2 (60 Gy)				
D_max_ (Gy)	64.88 ± 0.66	65.04 ± 0.70	−0.806	0.430
D_mean_ (Gy)	62.36 ± 0.53	62.41 ± 0.56	−0.287	0.777
CI	1.055 ± 0.049	1.039 ± 0.047	1.643	0.117
HI	0.082 ± 0.016	0.089 ± 0.016	−1.646	0.116

**Table 5:** **Comparison of OAR sparing between IMRT and IMRT_PMC&CST**

	**IMRT (Gy)**	**IMRT_PMC&CST (Gy)**	***t***	***p***
Ipsilateral lens D_max_	2.67 ± 1.67	2.76 ± 1.64	−1.574	0.132
Ipsilateral lens D_mean_	1.98 ± 1.45	2.03 ± 1.50	−1.294	0.211
Contralateral lens D_max_	2.38 ± 1.97	2.43 ± 1.95	−1.063	0.126
Contralateral lens D_mean_	1.84 ± 1.69	1.89 ± 1.72	−0.940	0.359
Ipsilateral optic nerve D_max_	12.32 ± 13.38	12.44 ± 13.91	−0.599	0.556
Ipsilateral optic nerve D_mean_	8.57 ± 9.40	8.78 ± 10.08	−1.126	0.274
Contralateral optic nerve D_max_	7.02 ± 7.68	7.07 ± 7.71	−0.353	0.728
Contralateral optic nerve D_mean_	4.59 ± 4.76	4.66 ± 4.95	−0.729	0.475
Optic chiasm D_max_	12.76 ± 13.53	12.49 ± 13.20	1.488	0.153
Optic chiasm D_mean_	8.28 ± 8.70	8.20 ± 8.63	0.573	0.573
Brainstem D_max_	14.04 ± 11.62	13.56 ± 11.08	1.260	0.223
Brainstem D_mean_	6.52 ± 6.99	6.46 ± 7.05	0.790	0.439

**Table 6:** **Comparion of radiation doses betweeen IMRT and IMRT_PMC&CST**

	**IMRT (Gy)**	**IMRT_PMC&CST (Gy)**	***t***	***p***
Ipsilateral PMC D_max_	46.54 ± 7.77	33.20 ± 11.13	7.304	<0.001
Ipsilateral PMC D_mean_	27.67 ± 8.06	19.99 ± 8.78	7.150	<0.001
Contralateral PMC D_max_	21.40 ± 10.94	16.16 ± 9.07	5.250	<0.001
Contralateral PMC D_mean_	14.11 ± 7.57	9.82 ± 5.62	5.276	<0.001
Ipsilateral CST D_max_	50.61 ± 4.72	40.37 ± 6.55	9.233	<0.001
Ipsilateral CST D_mean_	36.06 ± 7.57	27.72 ± 8.87	8.032	<0.001
Contralateral CST D_max_	36.11 ± 10.05	22.52 ± 10.36	6.959	<0.001
Contralateral CST D_mean_	18.90 ± 7.45	12.59 ± 5.51	6.362	<0.001

## Discussion

Radiation therapy is commonly applied to the brain tumors due to its ability to control cell growth; however, radiation therapy can have detrimental effects on the central nervous system causing neurological complications. The response of cerebral tissue to radiation can lead to the deficits in neural functions [9,10]. The extent of neurologic deficit is associated with the location and size of radiation-induced brain injury [11,12]. Efforts dedicated to the precise division of brain lesions have been made to reduce the risk of neurological complications caused by the radiation therapy.

The DTI and BOLD-fMRI have recently been used to identify the white-matter pathways and functional structures of the brain. In our previous study, we proposed a clinically feasible protocol of integrating BOLD-fMRI and DTI to optimize the extent of resection involving the cortical motor areas and subcortical white matter tracts in patients with brain gliomas. Those information helped neurosurgeons resected the maximum amount of tumor while still preserving the most critical cortices of the brain, thus resulting in enhanced postoperative quality of life for patients [13]. The incorporation of this information for radiosurgery planning has also been suggested. Liu et al. has reported a novel method to integrate the fMRI brain activation map with treatment planning for stereotactic radiosurgery (SRS). Direct irradiation of the eloquent cortices was avoided by multiple radiation arcs or static radiation beams in SRS planning, and the average dose reduction to the eloquent cortices was 32% [14]. In addition, it has been reported that the risk of radiation-induced neuropathy was minimized by the integration of tractography of the brain white matter with DTI into radiation treatment planning of radiosurgery using Gamma Knife [15]. Moreover, Pantelis et al. has demonstrated that critical structures of brain could be marked and spared with the aid of the integration of BOLD-fMRI and DTI into CyberKnife stereotactic radiosurgery [16].

In this study, BOLD-fMRI and DTI were used to localize the bilateral PMCs and CSTs and the information obtained from these two technologies were integrated into radiation treatment planning. The first part of our study (3DCRT versus IMRT) indicated that there was no significant reduction in the dose to bilateral PMCs and CSTs between the 3DCRT and IMRT plans. The critical structures adjacent to the target volume marked as OARs can be better spared during the IMRT planning process due to a steep dose gradient and a high conformity [17,18]. The second part of our study (IMRT versus IMRT_ PMC&CST), has shown that a significant reduction in the dose to bilateral PMCs and CSTs regions can be achieved without compromising the coverage of planning target volume and the limiting dose to these conventional OARs.

Sparing of the bilateral PMCs and CSTs does not represent any significant breakthrough in the treatment of brain tumors, but we have demonstrated that it is feasible to reduce the irradiation of critical structures adjacent to the target volume. The development of the most appropriate IMRT plan for the patient could be achieved by the identification of the important functional structures of the brain tissues proximal to the tumors. Sparing these vital functional structures is important to maintain quality of life, even in those patients with restricted life expectancy. The current study has shown that the DTI examination and MR Spectroscopy are valuable tools to differentiate the postoperative recurrent glioma from the radiation injury for patients with a glioma [19,20].

We are currently investigating paradigms for Broca’s and Wernicke’s areas (speech center), Broadmann-17 functional structures (visual center) and optic tracts. The application of the fMRI in low grade cases has also been validated.

## Conclusions

In conclusion, integration of BOLD-fMRI and DTI into radiation treatment planning is feasible and beneficial. With the assistance of the above-described techniques, the structures adjacent to suspicious cancerous lesions could be clearly marked as OARs and spared during treatment. However, a wider investigation and the longer-term clinical follow up are required to further validate the effect of the integration of BOLD-fMRI and DTI on sparing normal tissues.

## Abbreviations

BOLD-fMRI: Blood oxygen level dependent functional magnetic resonance imaging
DTI: Diffusion tensor imaging
3DCRT: Three-dimensional conformal radiation treatment
PMCs: Primary motor cortexes
CSTs: Corticospinal tracts
CT: Computed tomography
OARs: Organs at risk
IMRT: Intensity-modulated radiation therapy
IMRT_ PMC&CST: Intensity-modulated radiotherapy planning with PMC and CST information
WHO: World Health Organization
MRI: Magnetic resonance imaging
SE-EPI: Spin echo-echo planar imaging
ROI: Region of interest
GTV: Gross tumor volume
CTV1: Initial clinical target volume
PTV1: Initial planning target volume
CTV2: Second clinical target volume
PTV2: Second planning target volume
DVHs: Dose volume histograms
Dmax: The maximum dose
Dmean: Mean dose
CI: Conformity index
HI: Homogeneity index
